# Tailoring medical education to meet speciality needs

**DOI:** 10.1007/s40037-014-0131-2

**Published:** 2014-06-18

**Authors:** Sayinthen Vivekanantham, Rahul Ravindran, Dushyanth Gnanappiragasam

**Affiliations:** Imperial College School of Medicine, Imperial College London, London, SW7 2AZ UK

We read Borges et al. [1] and Goodin et al. [2] articles with interest in the context of considering the factors that effect speciality choice. Goodin et al. conclude that enriching a student’s calling in relation to speciality choice is vital to those who struggle with choosing a speciality. With certain specialities currently facing shortages, such as psychiatry, care of the elderly and emergency medicine, this remains an important issue in both developed and developing countries. New strategies to tackle this discrepancy are essential to minimizing this dawning health burden.

Speciality choice determinants are multifactorial, with personality traits, medical school experiences, social reward and work-life balance forming important components; however, having role models is widely recognized as the single most important predictor [3]. Furthermore, faculty composition and curricula components also predict speciality choice [4], perhaps by creating further opportunity to acquire role models within specialties. With certain specialities facing shortages, we first propose that dynamic redistribution of curricular components may be one solution. Curricular ratios of exposure to different specialities could be tailored to the speciality needs within a particular location, increasing and decreasing exposure to undersubscribed and oversubscribed specialities respectively. Secondly, undersubscribed speciality placements should be positioned earlier within the medical curriculum, as this has been shown to increase speciality interest [5].

We also welcome innovative programmes to increase students’ exposure to undersubscribed specialities, and hence create opportunities to develop role models. These may include encouraging the formation of extracurricular interest groups and speciality taster weekends. Indeed, a child and adolescent psychiatry educational initiative was implemented at the Mayo Clinic to deliver an interactive lecture series via teleconferencing to other US medical schools. This was demonstrated to instigate favourable opinions towards the speciality among students when asked about their career path [6].

Such strategies are of critical importance in developing countries. If workforces are drawn to specialities of interest within their home country, health professionals may be more inclined to practice in their country of origin. Recently, 57 % of students in Malawi reported an interest in pursuing specialities currently not offered in Malawi itself [7]. The strategies outlined may play an important role in curtailing the currently recognized ‘brain drain’ from these regions.
